# Initial evenness determines diversity and cell density dynamics in synthetic microbial ecosystems

**DOI:** 10.1038/s41598-017-18668-1

**Published:** 2018-01-10

**Authors:** Elham Ehsani, Emma Hernandez-Sanabria, Frederiek-Maarten Kerckhof, Ruben Props, Ramiro Vilchez-Vargas, Marius Vital, Dietmar H. Pieper, Nico Boon

**Affiliations:** 1 Center for Microbial Ecology and Technology (CMET), Coupure Links 653, 9000 Ghent, Belgium; 2 Microbial Interactions and Processes Research Group, Helmholtz Centre for Infection Research, Inhoffenstr. 7, Braunschweig, 38124 Germany

## Abstract

The effect of initial evenness on the temporal trajectory of synthetic communities in comprehensive, low-volume microcosm studies remains unknown. We used flow cytometric fingerprinting and 16S rRNA gene amplicon sequencing to assess the impact of time on community structure in one hundred synthetic ecosystems of fixed richness but varying initial evenness. Both methodologies uncovered a similar reduction in diversity within synthetic communities of medium and high initial evenness classes. However, the results of amplicon sequencing showed that there were no significant differences between and within the communities in all evenness groups at the end of the experiment. Nevertheless, initial evenness significantly impacted the cell density of the community after five medium transfers. Highly even communities retained the highest cell densities at the end of the experiment. The relative abundances of individual species could be associated to particular evenness groups, suggesting that their presence was dependent on the initial evenness of the synthetic community. Our results reveal that using synthetic communities for testing ecological hypotheses requires prior assessment of initial evenness, as it impacts temporal dynamics.

## Introduction

Microbial communities, where cells interact and communicate with one another and influence each other’s behaviour, are dynamic change agents in numerous ecosystems^1,2^. Comprehensive understanding of community properties such as diversity and structure is currently insufficient and, consequently, the description of natural communities remains challenging^3,4^. Uncovering functional and/or active members of natural microbial communities is a complex task. Hence, model systems have been developed. Synthetic communities are simplified representations of natural ecosystems, with improved reproducibility^5^ in a controlled environment^6,7^. These have been applied to study microbial interactions and biodiversity-production relationships^8,9^. For instance, for bioethanol production^10,11^, bioremediation of contaminated areas^12^, recycling of waste products during long distance space exploration^13^, and as an alternative for human faecal transplants^14^.

The impact of diversity, richness, and evenness on ecosystem functions such as stress resistance, invasion, and predation interactions^15–17^ has been reported. Although evenness influences community dynamics^18,19^, the stability of synthetic communities with different initial evenness over time is yet to be elucidated. In this study, we monitored the progress of communities with different initial evenness, and we hypothesized that these communities evolve in a similar and simultaneous fashion. A previous report showed that initial community evenness is a key factor for preserving the stability of an ecosystem^17^. Hence, extensive characterization of the evolving community structure is essential for a comprehensive overview of this process.

Currently, microbial community structure and dynamics are often determined based on 16S rRNA gene amplicon sequencing, which remains the standard method for culture-independent surveys of microbial diversity. On the other hand, flow cytometry (FCM) couples high accuracy with sensitivity, ranging from a single cell level to the community level^20^. FCM is a fast, high throughput method with a wide variety of potential applications, particularly in medical research and microbial ecology. Nonetheless, it is mostly limited to liquid samples and only partial information on community structure can be obtained from this analysis^21^. Flow cytometry fingerprinting (FCFP) is a promising approach to monitor complex microbial communities and to detect changes in the structure of communities. FCFP was applied to estimate the biodiversity of microbial communities, using a phenotypic diversity index based on single cell phenotypic characteristics, such as morphology and nucleic acid content^20^, but has not been used in synthetic communities previously. Additionally, FCM and sequencing techniques have been applied together to gain insight into community structure and dynamics over time^22–28^. In contrast with these studies, which surveyed natural communities, we monitored the temporal trajectory of synthetic communities. For these reasons, we employed complementary techniques to survey the community evolution of synthetic communities.

In this study, we assembled 100 different synthetic communities with the same richness but different initial evenness. We then combined flow cytometric fingerprinting and amplicon sequencing of the 16S rRNA gene, to monitor how initial evenness variations induced different temporal dynamics in community structure at the taxonomic and physiological level. Understanding these variations can assist us in recognising critical time points for community stability and resilience, which may be potentially modulated in synthetic microbial communities.

## Results

### Phenotypic diversity and total cell counts fluctuate over time

**Figure 1:**
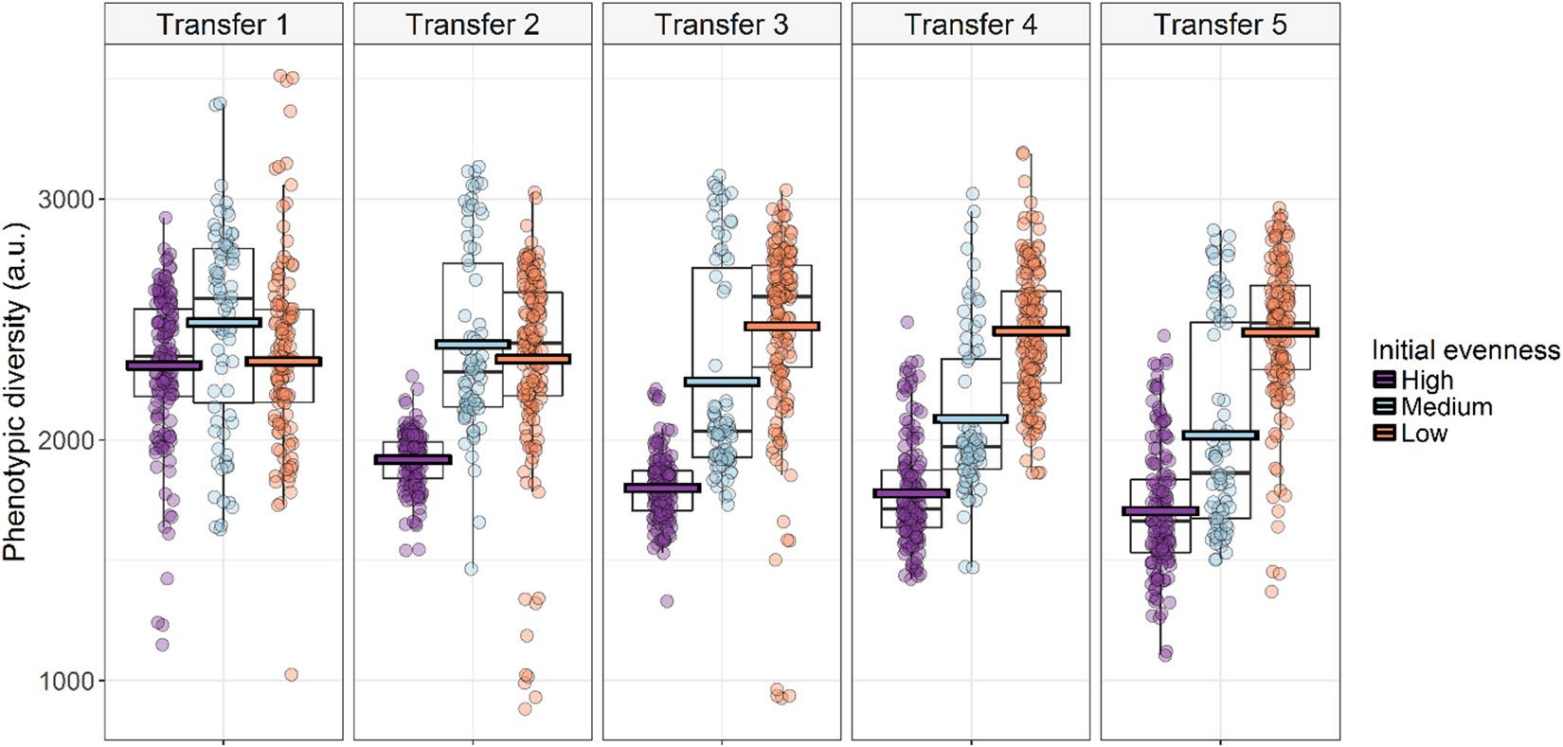
Phenotypic diversity (D_2_) dynamics for all microbial communities between transfer 1 (48 h) to 5 (240 h), calculated based on the flow cytometry data (n = 1913). Colored horizontal bars indicate the predicted mean from the generalized linear mixed model.

**Figure 2:**
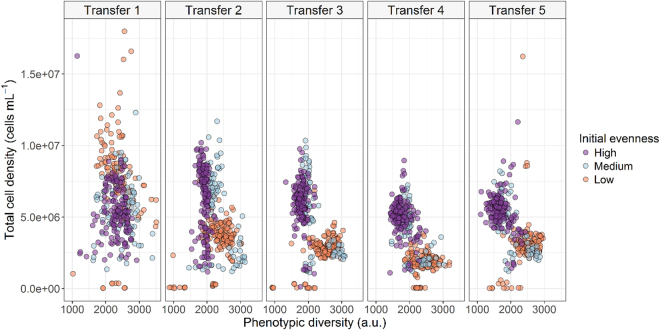
Scatterplot of the total cell density vs. the phenotypic diversity (D_2_, inverse Simpson index) at the end of each subsequent transfer following the initial inoculation.

Shifts in community structure were monitored in one hundred microcosms with same richness but varying initial evenness (divided into low, medium or high evenness group). Each synthetic community was sampled before a medium transfer that occurred at five discrete time points (at 48 h, 96 h, 144 h, 192 h, and 240 h). A transfer consisted of inoculating five percent of the liquid microcosm into fresh medium, (48 h). A total of 1913 observations were recorded and a significant decrease in the phenotypic diversity (Hill order 2) (P < 0.001) in high and medium evenness groups was observed, following flow cytometric fingerprinting. Conversely, there was no significant difference (P = 0.18) in the low evenness group (Fig. 1 and Supplementary Figure S1). Cell numbers and phenotypic diversity were measured at the end of each subsequent transfer (Fig. 2). The total cell count was not significantly different between medium and high evenness categories, but both groups differed in total cell counts with the low evenness category at transfer 1. Communities with high initial evenness separated from low and medium evenness groups, and showed higher total cell count and less diversity. Furthermore, mean phenotypic diversity and total cell count were significantly different at transfer 5 between all evenness groups (P < 0.0001, Supplementary Table S1).

### Community structure converged regardless of initial evenness

16S rRNA gene amplicon sequencing was performed to validate the phenotypic diversity trends revealed by flow cytometry, and to quantify each member of the synthetic communities at the start of the experiment (transfer 0) and after transfer 5.

**Figure 3:**
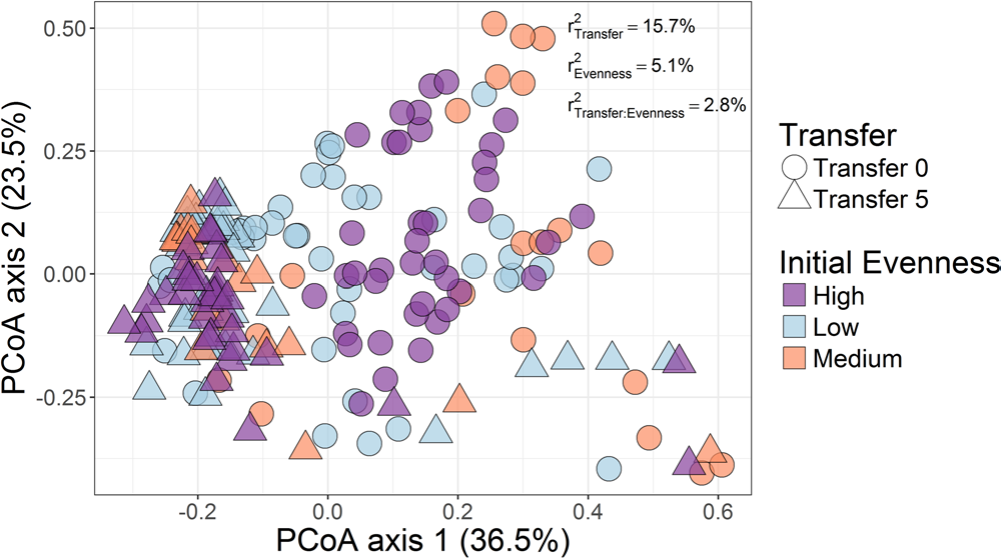
Principal coordinate analysis (PCoA) indicating differences in community structure between transfers 0 (0 h) and 5 (240 h). The variance explained by the experimental factors (P < 0.01) is indicated on the top right (PERMANOVA).

Several diversity indices were utilized to describe community composition, structure, and dynamics from the sequencing data. We applied the Fisher’s alpha diversity, Shannon–Wiener index, Pielou’s Evenness, Simpson’s, and inverse Simpson indices in this experiment. All diversity indices excepting for Simpson index were significantly different between transfer 0 and 5. The decrease in diversity was significant in all evenness groups across transfers (*P* < 0.05, Table 1). The evenness showed a decreasing trend between the start and end of the experiment, and the difference was significant (*P* < 0.005) in communities with high initial evenness. The results of different diversity indices were confirmed by measuring three Hill orders (Supplementary Figure S2). *Delftia*, *Aeromonas*, *Serratia* and *Bacillus* were the most abundant bacteria at transfer 5 in all evenness groups. The relative abundances of *Enterococcus* and *Clostridium* were significantly higher in the Low initial evenness group, in comparison with those in the Medium and High evenness groups (Supplementary Figure S3 and Supplementary Table S2). Beta diversity analysis through non-metric multidimensional scaling (nMDS) showed that communities tended to evolve towards the same structure at transfer 5 (Fig. 3). Moreover, permutational multivariate analysis of variance (PERMANOVA) confirmed that evenness did not explain the differences in community composition at the final transfer (*R* ^2^ = 0.03791, *P* = 0.108). These observations confirmed that alpha and beta diversity were not significantly different within and between the communities of different initial evenness.

**Table 1:** Differences in diversity and evenness indices between transfers 0 (0 h) and 5 (240 h). Different superscripts within column indicate significantly different means (n = 100).

Evenness	Transfer	Mean					
		Pielou	Shannon	Simpson	Fisher’s Alpha	Inverse Simpson	Total species
High	0	0.68^a^	1.56^a^	0.71^a^	1.25^a^	3.81^a^	10
Medium	0	0.55^b^	1.24^b^	0.60^bc^	1.20^ab^	2.81^bc^	10
Low	0	0.60^b^	1.32^b^	0.65^b^	1.14^b^	3.18^b^	9
High	5	0.57^cb^	1.03^cb^	0.54^c^	0.73^c^	2.30^d^	6
Medium	5	0.58^cb^	1.05^c^	0.56^c^	0.73^c^	2.35^cd^	6
Low	5	0.57^cb^	1.03^c^	0.55^c^	0.74^c^	2.35^cd^	6
*P* value							
Evenness		0.03	0.006	0.16	0.05	0.008	0.06
Transfer		0.06	<0.0001	<0.0001	<0.0001	<0.0001	<0.0001
Evenness*Transfer		0.03	0.003	0.02	0.001	0.002	0.002

Multiple factor analysis (MFA) further explained how the diversity and evenness metrics were associated with the relative abundance of each member of the synthetic community (supplementary Figure S4). Each of the dimensions of the MFA describes the continuous and categorical variables included in the analysis. In our case, the continuous variables were the relative abundances of the bacterial species, and the categorical variable was the initial evenness category. In this way, one categorical variable and continuous variables will compose each dimension. For each categorical factor level (evenness category), a one-way analysis of variance was performed using the coordinates of the samples on the axis belonging to either Low, Medium or High evenness. Then, for each factor level of a category (i.e. Low, Medium or High), a Hotelling T^2^ -test was used to compare the average of category with the general average. For instance, the distance from the data point representing the abundance of strain A on the Low evenness category to the corresponding axis was calculated. This distance was compared to the distance from the data point representing the average coordinates of the relative abundance of strain A in the three evenness categories to the same axis. The *P* value associated to this test was transformed to a normal quantile to assess whether the mean of the category was significantly less or greater than 0. Negative values indicate negative correlations^29^.

Dimension 1 explained the 28% of the variance, while dimension 2 described 24% of the variance and dimension 3, nearly 14% (Supplementary Table S3). None of these dimensions was significantly associated with either of the three initial evenness categories. However, Dimension 4 explained the 10% of the total variance among microbial communities and included the relative abundances (continuous variable) of species composing communities with high initial evenness (categorical variable, Supplementary Table S3). In this dimension, richness and Fisher diversity index were positively associated between each other (*P* < 0.0001), and both were negatively associated with the relative abundances of *Rhizobium* and *Burkholderia* (*P* < 0.01). Fisher’s alpha diversity is an indicator for logarithmic changes in relative abundances. The positive association between richness and Fisher’s index indicated that high evenness communities tended to have high richness, but the distribution of these species was skewed. *Enterococcus*, *Aeromonas* and *Clostridium* were the main genera associated with dimension 4, potentially indicating that their relative abundances were associated with communities of high evenness. *Delftia* and *Tissierella* were associated with dimension 5, and therefore, with communities of low evenness.

## Discussion

### Effect of initial evenness on diversity and cell density dynamics

We monitored how stability and structure of communities with different initial evenness was impacted over time. Synthetic communities with fixed richness but different evenness were assembled and monitored by both 16S rRNA gene sequencing, to assess taxonomic diversity, and FCFP, to evaluate the phenotypic diversity. Changes in community dynamics as well as reduction in diversity were observed over time. Sequencing results revealed a reduction in diversity at transfer 5 relative to transfer 1, leading to equal α-and β-diversity at the final transfer regardless of the initial evenness. However, FCFP indicated that the decrease in diversity was only significant for the initial High and Medium evenness classes. In contrast with the sequencing results, FCM suggested that diversity may become similar but not identical over time.

Environmental filtering occurs when species are unable to tolerate environmental conditions. Based on this concept, the environment acts as a selective force which has effects on the distribution of biodiversity over the world^30^. The observed changes in evenness in our synthetic communities can also be a result of the microenvironmental conditions in the context of this experiment. Moreover, diversification continues until the maximum number of species that the ecosystem can support (carrying capacity of ecosystem) is reached. When biodiversity attains saturation, interactions can steer elimination of some taxa^31^.

As previously described, FCFP combined with multidimensional and statistical analysis have been used to detect changes in community composition of drinking water^32,33^. These previously reported results were based on natural communities, but in our experiment, we worked with small synthetic communities. Thus, our communities may not follow the same underlying ecological principles of natural communities, as a result of the synthetic nature of the experiment^34^.

Dissimilarities observed between the results of FCFP and amplicon sequencing may be as well due to inherent differences among the techniques: FCFP is based on staining nucleic acids of cells in suspension, whereas amplicon sequencing applications focus on rRNA gene hypervariable regions, and some limitations have been found in both techniques. Sequencing errors, difficulties in assessing operational taxonomic units (OTUs) and numerous 16S rRNA gene copies in some species are limitations of 16S rRNA techniques^35–37^. The phenotypic diversity estimation in our synthetic communities was based on optical features detected by flow cytometry, enabling us to describe the diversity of the synthetic communities across phenotypic characteristics (i.e. morphology and nucleic acid content), which cannot be inferred from molecular techniques. Recent research has shown strong correlation between the phenotypic and taxonomic diversity of natural freshwater communities in both low and high diversity environments^20,38^. In contrast to the sequencing-based approach, FCFP is fast (<1 h), cheap (<€1 sample^−1^) and requires only minute sample volumes (<1 mL) thereby allowing high-frequency and non-invasive tracking of sensitive biological processes, such as the feeding of invasive mussel species on bacterioplankton communities^38^.

**Figure 4:**
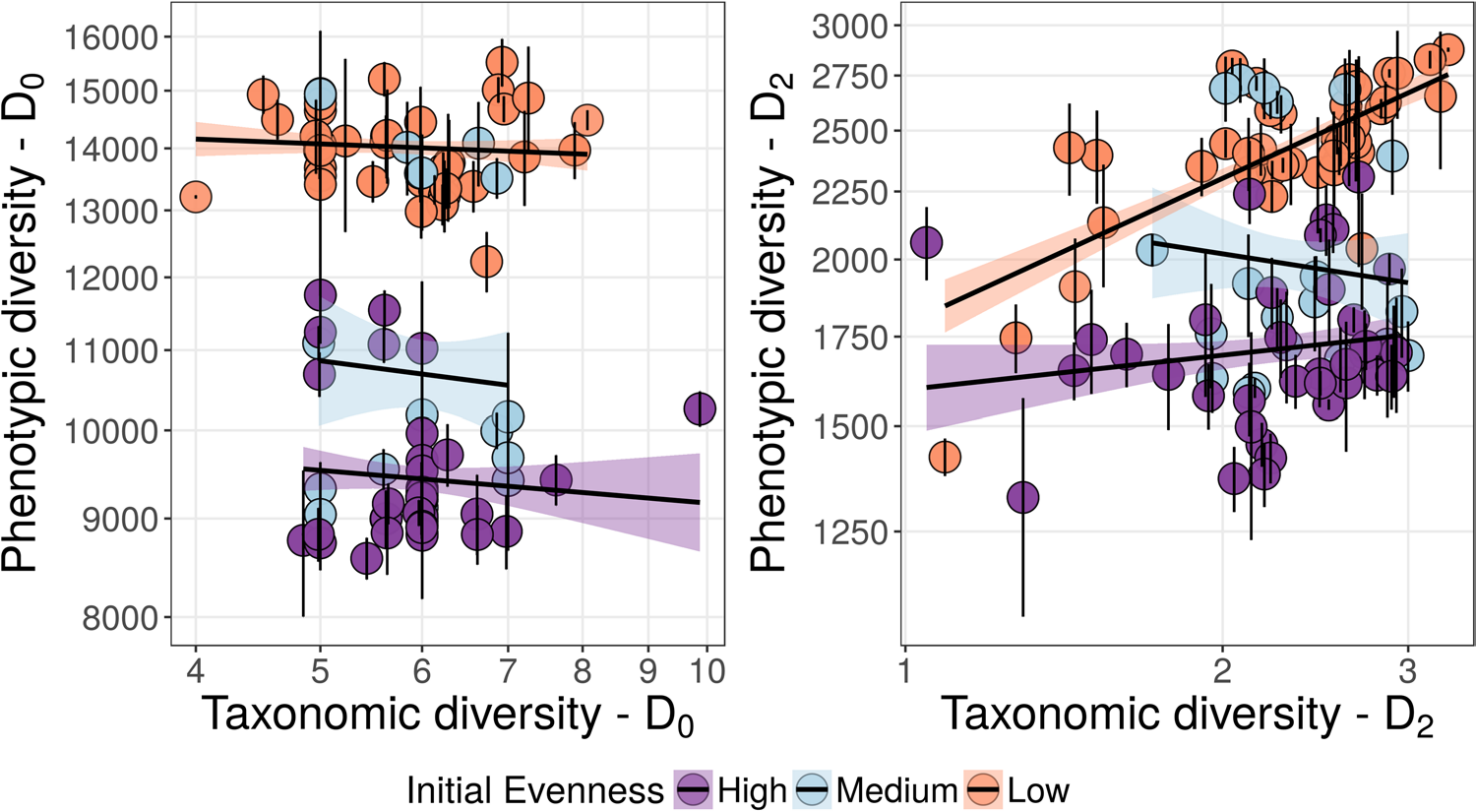
Correlation between D_0_ and D_2_ of the phenotypic and taxonomic diversity. Flow cyctometric fingerprinting diversity as opposed to taxonomic diversity based on diversity order 0 (left panel) and flow cyctometric fingerprinting diversity as compared to taxonomic diversity diversity based on diversity order 2 (right panel) at transfer 5.

However, further validation of FCM assessment of biodiversity in additional natural and synthetic environments is required^20^. In contrast to previous studies, we did not find a strong correlation between D_0_ and D_2_ of the phenotypic and taxonomic diversity for synthetic microbial communities (Fig. 4). FCFP successfully tracked changes between evenness classes in comparison with 16S amplicons sequencing at transfer 5. 16S amplicon sequencing is a standard metric of diversity in the field but it was not suitable for quantifying the relative species abundance in our system. Any PCR-based sequencing method is prone to biases when quantifying diversity, as result of extraction, PCR, issues during annotation, among others. This became evident when comparing the theoretical initial diversity with the 16S rRNA gene amplicon sequencing diversity assessment (Supplementary Figure S5). Our findings are however, increasingly being reported in literature, for example, a recent study described difficulties associated with correcting abundance biases in 16S amplicon data sets^39^.

Moreover, FCFP showed that the phenotypic diversity tended to be low when bacterial cell numbers were high in all our evenness groups. Thus, communities with low and medium initial evenness were more diverse and had lower total cell counts than communities with high initial evenness. Communities with high initial evenness showed higher cells numbers but low diversity. Milici *et al*. reported a negative correlation between diversity and bacterial cell numbers, suggesting that this may be a result of transient changes in environmental conditions^40^. Our results are also in agreement with the recently proposed hypothesis that microbes adhere to macro-ecological scaling laws, where evenness (diversity) decreases with increasing population size^41^. Zhang *et al*. implied that the probability of ecological drifts associated with birth and death, and fluctuations in relative population abundance decrease when there are smaller populations and lower growth rates, which may be the case in our synthetic communities. Thus, it was expected that diversity and bacterial abundance decreased concurrently^42^.

Based upon individual growth curves (data not shown), all strains should have reached stationary phase in between each transfer (i.e. <48 h). Single cells possess traits that increase group performance but that may be detrimental for individual cells^43^. For instance, fast growth may be inefficient, thereby impacting heterotrophic cell metabolism and ultimately entire population density^44^. In contrast, higher yields optimize limited environmental resources, and support the survival of the entire population^45^. Indeed, shifts between mixed and segregated cell lineages affect the evolution of cooperative and antagonistic phenotypes^46^. In natural settings, community members may differ in the level of cooperation, which makes social interaction open to exploitation^43^. For instance, *P*. *aeruginosa* delays investment into cooperation when its impact on fitness becomes negligible^47^.We artificially altered the fitness of certain strains by initially mixing the strains at different ratios. In this way, the differences in the evenness among communities may have triggered competitive interactions that ultimately drove the community to a low cell density. In addition, Zha *et al*. reported that evenness increased with increasing dispersal rates. It is possible that the repeated inoculation in our experiment may have had a similar effect^48^.

Our results showed a fluctuation of cell density over time in all communities. Thus, the effect of initial evenness on cell density after five subsequent transfers for the Low and Medium initial evenness categories was uncovered. Cell density decreased in all groups but communities with low initial evenness showed the fastest decrease over time. Communities with high initial evenness retained a higher cell density over time in comparison with other groups. The results at the final transfer in all evenness groups indicated that *Bacillus*, *Rhizobium* and *Burkholderia* had very low abundances in the Low evenness group, whereas *Rhizobium* and *Burkholderia* were almost absent in the Medium group. Therefore, higher cell numbers at the final transfer in the High evenness group may be due to the presence of all species, as indicated by the sequencing results.

### Implications of initial evenness for synthetic microbial community experiments

Knowledge of differences between communities is essential for identifying ecosystem dynamics. For instance, Wittebolle *et al*. proposed that communities with high initial evenness may present improved resistance during environmental oscillations, by creating specialized microcosms with varying evenness without changing richness. Communities with high initial evenness showed higher functional stability even under non-stressed conditions in comparison with those with low initial evenness^17^. Importantly, most synthetic community studies did not fully control for potentially evenness-dependent temporal effects on community diversity, function, and cell density. Understanding cooperative and competitive interactions assists to clarify community function, assembly, and stability. Collaborations within and among bacterial communities can arise when one or a group of species provide nutrition or protection to another, whereas competition occurs when some species compete for a shared metabolite^49^. In the context of this experiment, *Bacillus*, *Enterococcus*, *Aeromonas*, and *Clostridium* were associated with low evenness whereas *Delftia* and *Tissierella* were correlated with high evenness, suggesting that they are more abundant on either group. Therefore, *Delftia* and *Tissierella* thrived in our communities with High evenness, while *Aeromonas* and *Enterococcus* succeed in the communities designed with Low evenness. *Aeromonas* abundance was negatively correlated with *Delftia*, *Serratia* and *Enterococcus* abundance but positively correlated with *Bacillus* abundance. Thus, *Serratia* and *Enterococcus* were less present in communities with high relative abundance of *Aeromonas*. *Burkholderia* and *Rhizobium* were correlated with each other positively, which may explain their consistent low relative abundance in all the evenness groups.

Understanding how community structure changes over time is a fundamental question in ecology. The structure and composition of natural microbial communities remains to be elucidated because of the presence of non-cultivable species. The complete description of their functions and microbial interactions remains challenging. Unlike temporal dynamics of animal and plant communities, the temporal dynamics exhibited by microbial communities have not been fully described^50^. Based on our results, the combined use of FCM and sequencing provided an overview of community structure dynamics, allowing for efficient monitoring of natural microbial communities. Synthetic communities are of special interest, because description of their characteristics has not been performed yet. Knowledge of these traits will assist us to elucidate the effect of time on community stability and resilience, which are key functional characteristics of microbial communities. Using synthetic and controlled microbial communities may also further our mechanistic understanding and support the development of models with increased predictive power. In the context of our study, deterministic selection may have impacted the population size over time. This finding may be relevant for the development of engineered communities with targeted functionality, such as those in wastewater treatment or environmental bioremediation. By monitoring diversity, structure, resilience and stability of communities, bioremediation efficiency^51^ may be predicted, for instance, and successful *in situ* bioremediation techniques can be implemented^52^.

## Methods

### Construction of synthetic communities

A total of 10 environmental bacterial species from 4 phyla (Table 2) were mixed in different proportions to create microcosms with varying evenness but with the same number of species (richness). We quantified initial evenness using the Pielou index, which is an excellent measure for community structure. This metric encapsulates the component of species richness within an ecosystem, as well as the species distribution, and it is rescaled between 0 (most uneven community) and 1 (completely even)^53,54^. In this way, the synthetic communities were grouped in Low, Medium, and High evenness (between 0–0.3, 0.4–0.6 and 0.6–1 respectively), based on their initial Pielou evenness. One hundred synthetic communities were sampled from an experimental design of one million *in silico* simulated synthetic communities (supplementary information: experimental design section and Supplementary Table S4). The 100 different communities were distributed between the evenness classes: 40 microcosms with low, 20 with medium and 40 microcosms with high evenness. For this purpose, 96 deep well microtiter plates were filled with 2 ml of fresh LB medium and 5% (v/v) of the microcosms, with adjacent duplicates of each mixture. Subsequently, these plates were incubated at 28 °C on a microplate shaker at 250 rpm. After 48 h, 5% of the community was transferred to another microplate with fresh medium and incubated for 48 h. This procedure was repeated five times, i.e. for a total of 240 h. Cell number and community phenotypic diversity were assessed after each transfer (at 48 h, 96 h, 144 h, 192 h, and 240 h) by means of flow cytometry (Accuri C6 Flow cytometer, BD Biosciences, Erembodegem, Belgium), with SYBR green I staining of the nucleic acids as described by De Roy *et al*.^5^. Further, amplicon sequencing of the 16S rRNA genes (Illumina Miseq, Illumina, Hayward, CA, USA) was performed at transfer 0 and 5 to supplement the results of FCFP.

**Table 2:** Strains used to create the microcosms with different degrees of initial evenness. ^a^Draft genome is available^66^.

Phylum	ID	Isolated site	Closest taxonomic identifier (NCBI ID)
α Proteobacteria	*Rhizobium sp*.	soil & water contaminated with alkanes	*Rhizobium daejeonense*
β Proteobacteria	*Delftia sp*.	soil & water contaminated with alkanes	*Delftia acidovorans*
	*Burkholderia sp*.	soil & water contaminated with alkanes	*Burkholderia xenovorans*
γ Proteobacteria	*Aeromonas sp*.^a^	Sand filter	*Aeromonas sp*. *EERV15*
	*Serratia sp*.	soil & water contaminated with alkanes	*Serratia myotis*
Firmicutes	*Enterococcus sp*.	soil & water contaminated with alkanes	*Enterococcus qallinarum*
	*Bacillus sp*. (*Bacillus I*)	soil & water contaminated with alkanes	*Bacillus methylotrophicus*
	*Clostridium sp*.	soil & water contaminated with alkanes	*Clostridium bifermentans*
	*Tissierella sp*.	soil & water contaminated with alkanes	*Tissierella sp*. *FSAA-15*
	*Bacillus sp*.	soil & water contaminated with alkanes	*Bacillus mycoides*

### Cell number and phenotypic characteristics assessment

Synthetic communities were diluted (1,000, 5,000 or 10,000 times) in 145 mM of NaCl and stained with SYBR Green I to determine total cell count^5,55^. Four technical replicates of each sample were prepared and analysed to obtain robust estimates of the phenotypic characteristics. The data points belonging to the microbial community were isolated from (in-)organic and instrument noise using a gating strategy applied on the FL1-H and FL3-H channels (Supplementary Figure S6).

Microbial phenotypic diversity was estimated for all samples (n = 1913) using a novel computational method, which calculates various diversity estimators based on single-cell phenotypic features^20^. Nucleic acid content and scatter signals were employed to calculate the phenotypic diversity metrics (i.e. Hill numbers) from these distributions. We utilized the *flowBasis* and *Diversity* functions with default settings on the denoised data, per the protocol available at https://github.com/rprops/Phenoflow_package (v1.0, seed = 777). Samples with less than 1,000 cells were excluded from the analysis, resulting in a minimum sample size of 5,000 cells. We utilized the Hill diversity of order 2, also known as the inverse Simpson index, for inference on community diversity metrics.

### DNA extraction

One ml from each community was centrifuged 10 min at 13000 × g, supernatant was removed and the pellet was stored immediately at −20 °C until further analysis^56^. Cells were lysed with 1 mL of lysis buffer (100 mM Tris/HCl pH 8.0, 100 mM EDTA pH 8, 100 mM NaCl, 1% (m/v) polyvinylpyrrolidone and 2% (m/v) sodium dodecyl sulphate) and 200 mg of glass beads (0.11 mm, Sartorius), in a FastPrep^®^− 96 instrument (MP Biomedicals, Santa Ana, USA), two times for 40 s at 1600 rpm. After removing glass beads by centrifugation (5 min at 13000 g), DNA was extracted following a phenol–chloroform extraction. DNA was precipitated with 1 volume ice-cold isopropyl alcohol and 0.1 volume 3 M sodium acetate for 1 h at −20 °C. DNA pellet was dried and resuspended in 100 μL 1 × TE (10 mM Tris, 1 mM EDTA) buffer. Samples were immediately stored at −20 °C until further analysis. Quality of DNA was analysed by gel electrophoresis.

### 16 rRNA gene sequencing and analyses

The V1-V2 hypervariable regions of the 16 S rRNA gene were amplified as described by Camarinha-Silva *et al*.^57^. Sequencing was performed using Illumina MiSeq sequencer and reads were clustered allowing for two mismatches as previously defined^58^. The dataset was then filtered to consider only those phylotypes that were present in at least one sample at a relative abundance >0.1% or were present in all samples at a relative abundance >0.001%^57^. A total of 200 samples were analysed and a total of 6 million reads was obtained. Sequence composition was compared using the RDP Classifier tool^59^ and SILVA database^60^. After examining read counts, data were rarefied to a chosen maximum depth of 3317 sequences, using the phyloseq package from R^61^ and rarefaction curves were plotted using the vegan package in R^62^.

### Statistical analysis

Alpha diversity was assessed calculating the total number of species, Fisher’s diversity, Shannon, Simpson and inverse Simpson indices for all evenness categories (H, L or M) and for transfer, using the vegan software package in R^62^. Pielou’s index was used as indicator of evenness in the community. Differences in alpha diversity and evenness measures among transfers and evenness categories were compared using a repeated measures mixed model in SAS (version 9.3, SAS Institute, Cary, USA), and comparing multiple means using Tukey test. In this way, the differences in the diversity measures among communities could be attributed to either evenness category, transfer or to the interaction transfer*evenness category. Hill numbers or effective number of species^63^ were calculated from amplicon sequencing, as well as from FCM data, using the Phenoflow package. A generalized mixed model was constructed to evaluate the interaction effect between transfer and initial evenness on the phenotypic diversity (*nlme* package)^64^. Random intercepts were included for each biological replicate and autocorrelation was addressed by an AR1 structure. Parameters were estimated using the restricted maximum likelihood approach. Tukey’s all pair comparison was conducted for pairwise comparisons between initial evenness groups at each transfer. Normality and homoscedasticity of the model residuals were visually checked using diagnostic plots (Supplementary Figure S1).

Permutational multivariate analysis of variance (PERMANOVA) with 999 permutations was conducted to explore the percentage of variance that could be explained by the differences in beta diversity, based on Bray–Curtis distance matrices. Tukey’s test for pairwise comparisons of group mean dispersions was performed using the vegan package in R. Further, the function *adonis* was used to perform a one-way ANOVA, to determine the impact of evenness in community diversity. Differences in relative abundances of the bacterial genera composing each community were compared using a repeated measures mixed model in SAS, with the least squares means adjustment and Bonferroni correction for multiple comparisons^65^. Multiple factor analysis (MFA) was used to reveal whether any of the diversity metrics and/or evenness were associated with each member of the synthetic communities (FactoMineR package)^29^.

### Data availability

The authors declare that the main data supporting the findings of this study are available within the article and its Supplementary Information files. All FCS files of flow cytometry data, accompanying metadata have been deposited to the Flow Repository website with the repository ID FR-FCM-ZYBR (http://flowrepository.org/id/FR-FCM-ZYBR). The OTU table (97% identity) also has been added to supplementary information (Supplementary Table S5).

## Electronic supplementary material

Supplementary information
