# Supplementary material for: Initial evenness determines diversity and cell density dynamics in synthetic microbial ecosystems
Source: Sci Rep. 2018 Jan 10;8:340. doi: 10.1038/s41598-017-18668-1 (PMC5762898; doi:10.1038/s41598-017-18668-1)
Supplement: Supplementary file 1 — Supplementary information [file 41598_2017_18668_MOESM1_ESM.pdf]

SUPPLEMENTARY INFORMATION

For

Initial evenness determines diversity and cell density dynamics in Synthetic microbial ecosystems

Elham Ehsani[1], Emma Hernandez-Sanabria[1], Frederiek-Maarten Kerckhof[1], Ruben Props[1], Ramiro Vilchez-Vargas[1], Marius Vital[2], Dietmar H. Pieper[2] and Nico Boon[1*]

[1]Center for Microbial Ecology and Technology (CMET), Coupure Links 653, 9000 Gent, Belgium

[2]Microbial Interactions and Processes Research Group, Helmholtz Centre for Infection Research, Inhoffenstr. 7, Braunschweig 38124, Germany

[*] **Corresponding author:** Prof. Nico Boon, Center for Microbial Ecology and Technology (CMET), Department of Biochemical and Microbial Technology, Ghent University, Coupure Links 653, 9000 Gent, Belgium. Tel. (+32)9264 5976; Fax (+32) 9264 6248. Nico.Boon@UGent.be

Supplementary information: experimental design

To exclusively study the impact of initial evenness on community dynamics, communities of fixed richness were assembled, and other confounding factors were controlled as much as possible in constituting the Synthetic communities: only two pipetting volumes were chosen (400 and 800µL), and the initial concentration of each strain involved was set from 10^4 to 10^7 cells/mL with decimal increments. In total, 1 million Synthetic communities with differing evenness were simulated *in silico* (Figure E1), showing that there is no constant cell count that covers the entire evenness domain. However, in the data range indicated by the red box the cell count remained reasonably constant over the entire evenness domain. For reasons of feasibility, 100 data points were sampled from this simulated distribution by a block design (Figure E2) that assures that cell count and initial evenness were not correlated (Pearson r=-0.13, not significantly different from 0: p=0.19) . Finally it is clear that the simulated experimental design does not have confounding of the initial cell count with the initial evenness (Figure E3).

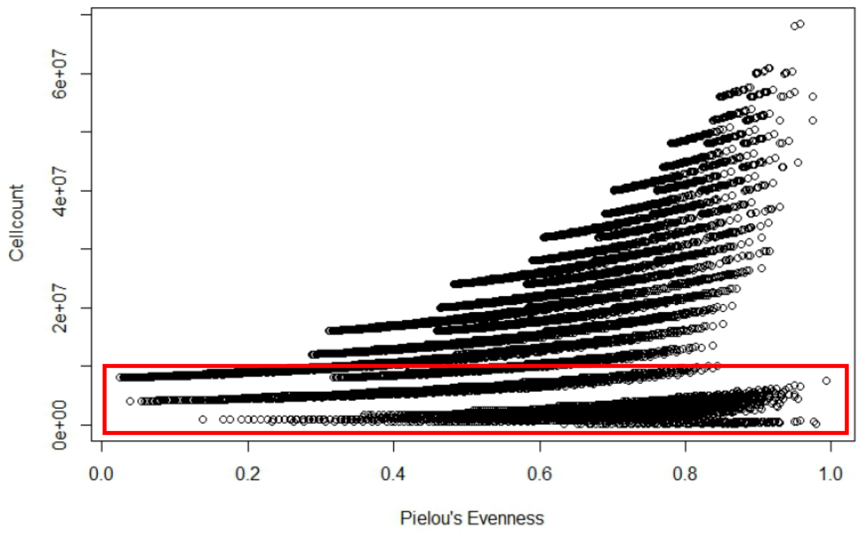

Supplementary experimental design Figure E1: Plot of Pielou evenness versus cell count for one million simulated Synthetic communities. The red box shows from which region the data was sampled (the region allowed for evenness and cell count to be uncorrelated).

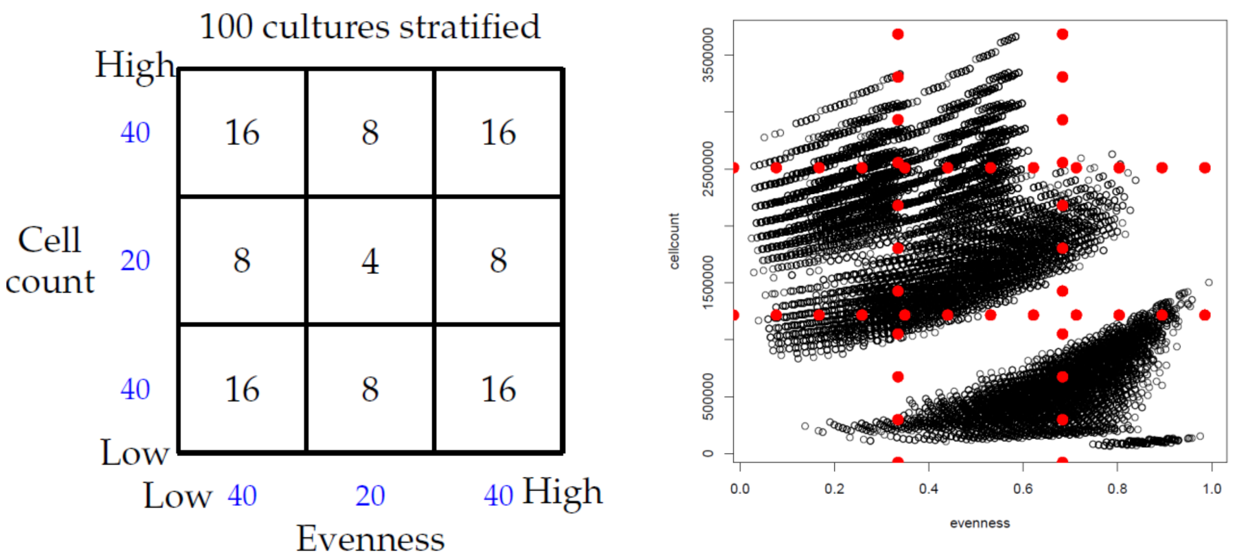

Supplementary experimental design Figure E2. Sampling strategy for Synthetic community assembly. The left panel shows number of community assemblies in each block with row- and column sums (blue). The right panel corresponds to the red box in Figure E1 and shows how the total simulation space was intersected using the block design to assure that evenness and cell count were not correlated.

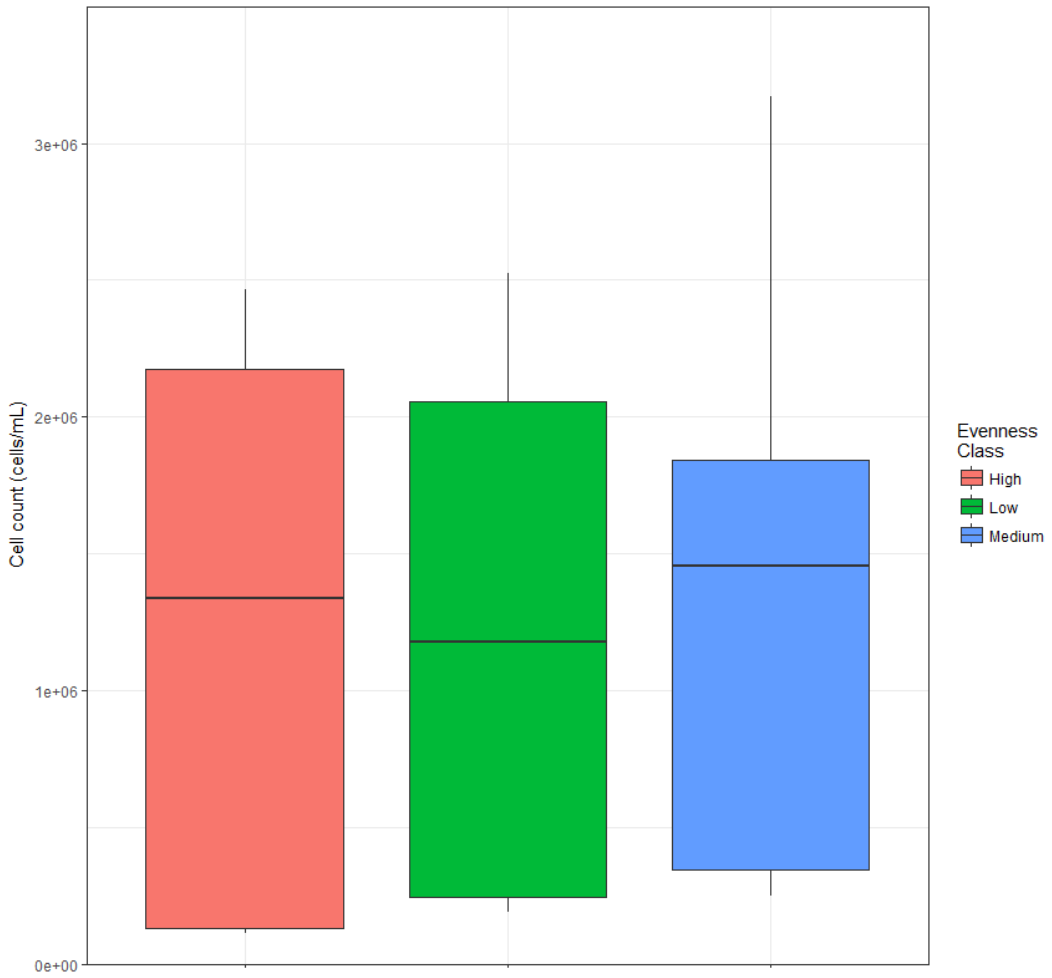

Supplementary experimental design Figure E3. Box-and-whisker plots for the initial cell count in each evenness class in the 100 sampled Synthetic communities from the experimental design.

Supplementary figures

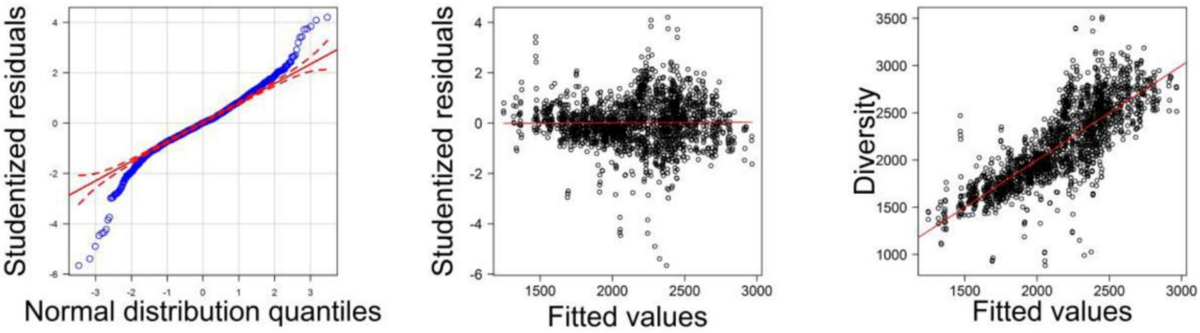

Supplementary Figure S1: Residual analysis for the linear mixed effects model used in Figure 1. Left panel: qqplot with 95% confidence intervals indicating an approximate normal distribution of the studentized residuals. Middle panel: slight heteroscedasticity present in the residuals, but REML parameter estimation is robust to this effect. Right panel: predicted vs. fitted values, red line indicates 1:1 ratio.

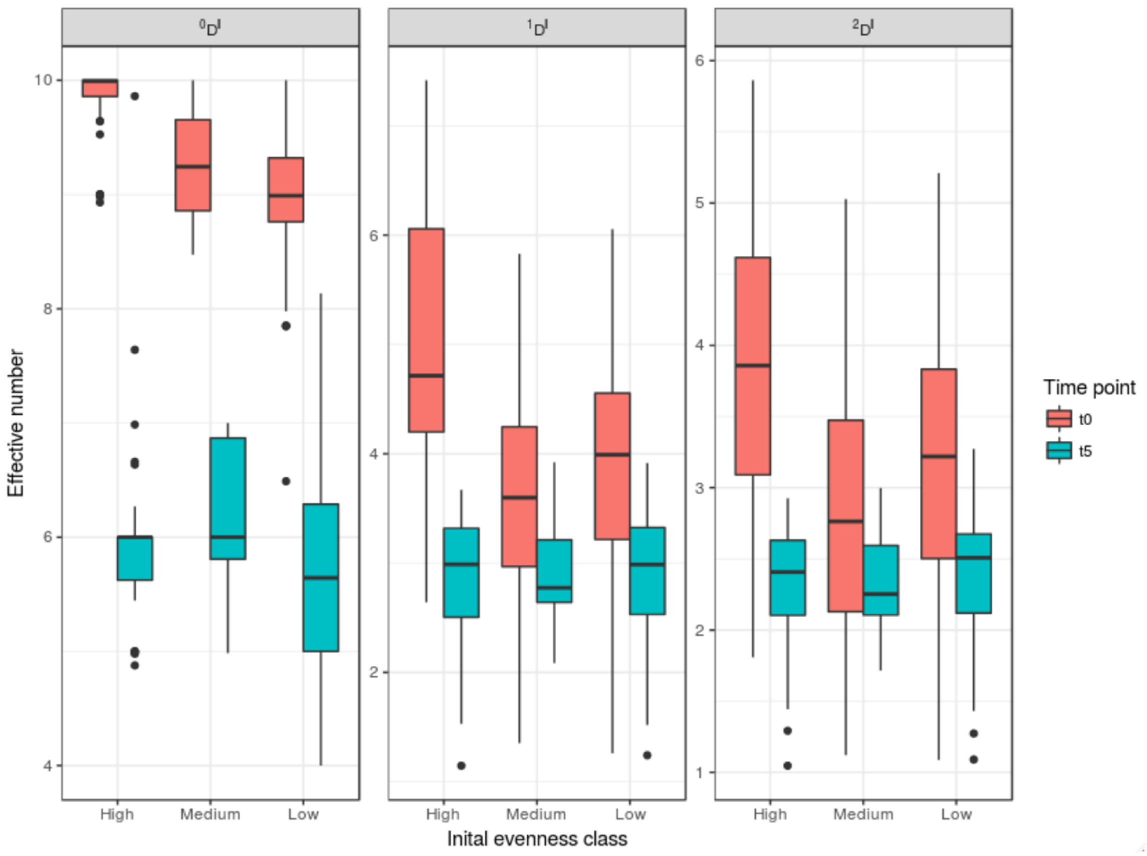

Supplementary Figure S2: The naive diversity of three Hill orders, $^{0}D^{I}$ (species richness), $^{1}D^{I}$ exp (Shannon entropy) and $^{2}D^{I}$ (Inverse Simpson), was measured in high, medium and low initial evenness groups at transfer 0 and 5 by using amplicon sequencing data. The results of Hill numbers were confirmed diversity reduction from transfer 0 to transfer 5 but no difference at final transfer in all evenness groups.

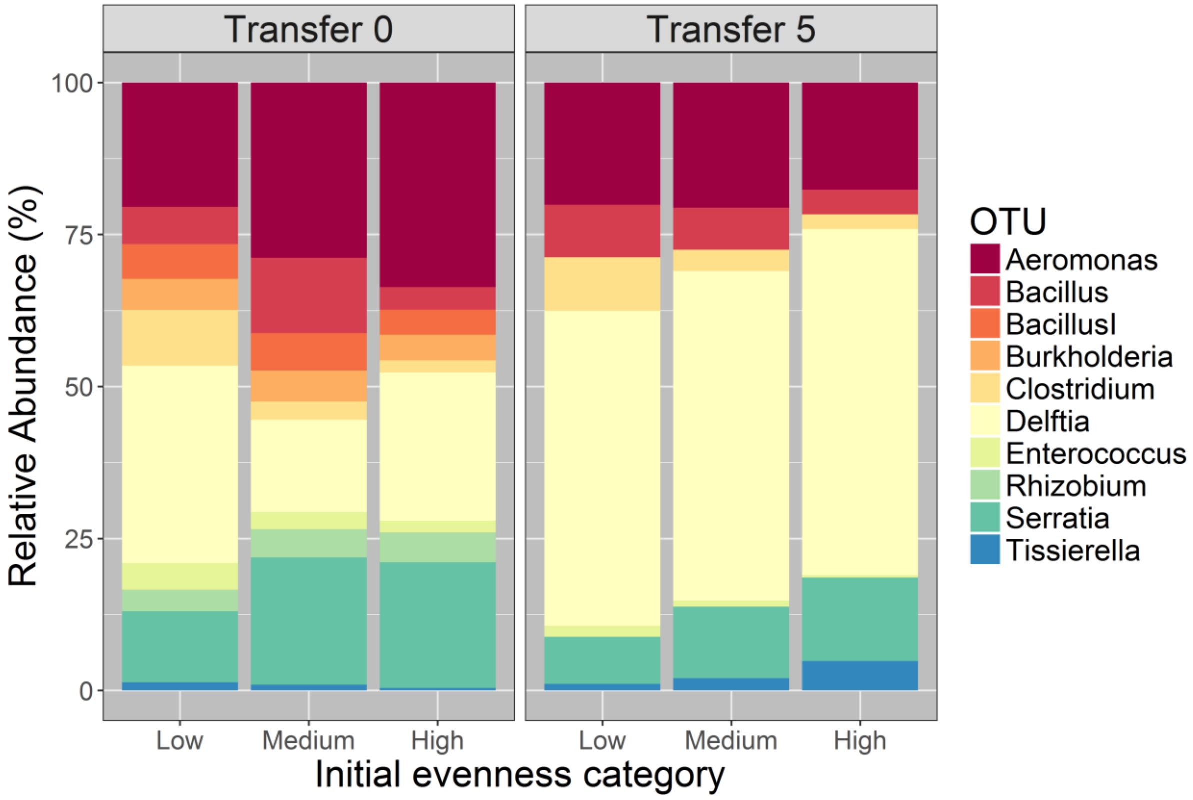

Supplementary Figure S3: Average community composition of the three evenness groups at transfer 0 and after transfer 5 (240h).

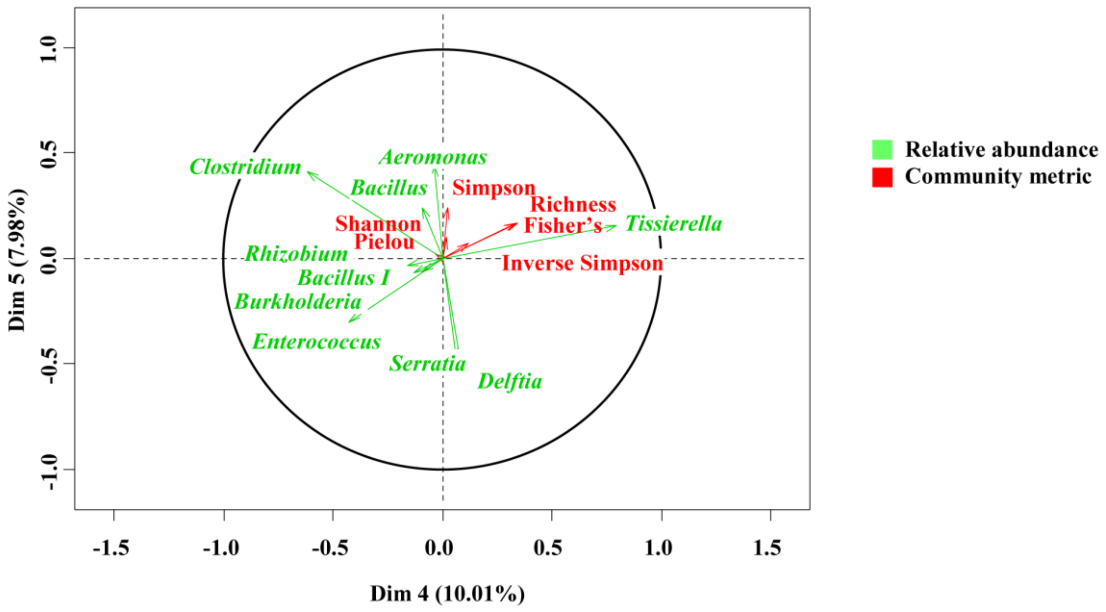

Supplementary Figure S4. Correlation circle indicates positive and negative correlation among variables. Longer vectors in the same direction indicate more positive correlations among variables, whereas longer vectors in the opposite direction indicate more negative ones.

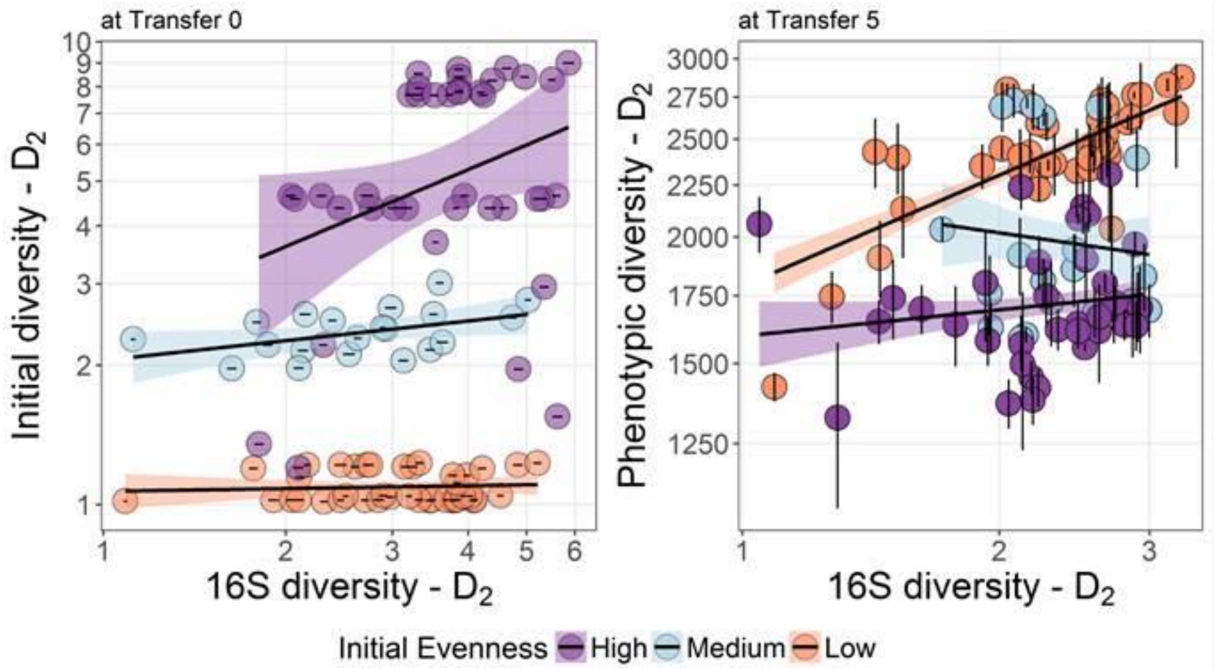

Supplementary Figure S5. Initial diversity of order 2 (effective number) as opposed to 16S rRNA gene amplicon sequencing diversity at transfer 0 (left panel) or flow cytometric fingerprinting diversity as compared to 16S rRNA gene amplicon sequencing diversity at transfer 5 (right panel).

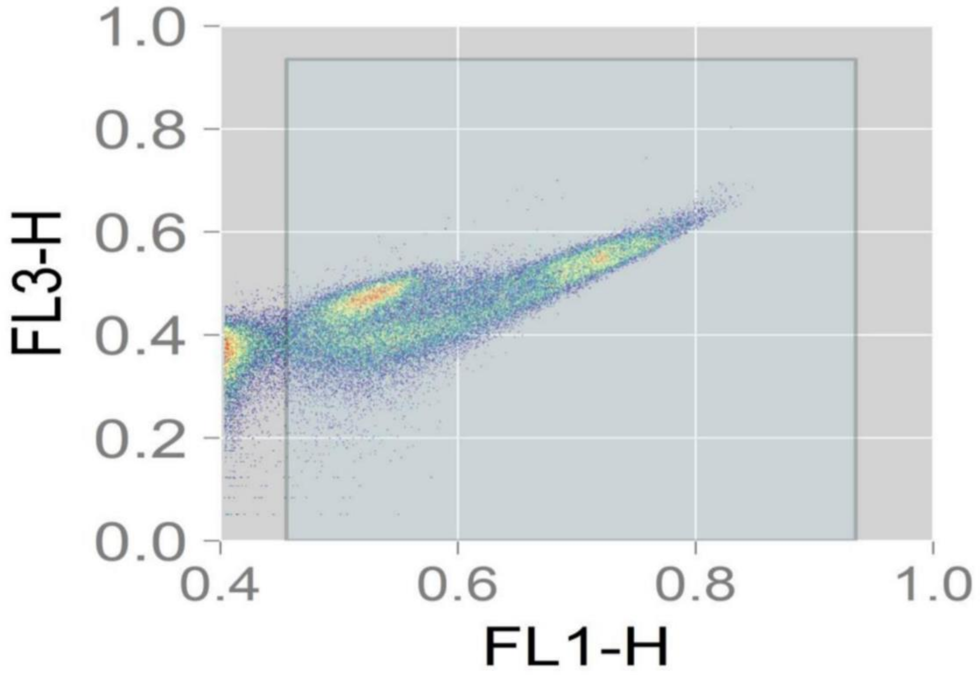

Supplementary Figure S6: Denoising strategy which was applied on all flow cytometry samples. Only signals that fell within the filter were retained for phenotypic diversity analysis. FL1-H and FL3-H were asinh-transformed and normalized to the [0,1] range

Supplementary tables

Supplementary Table S1: Diversity (inverse Simpson index) and total cell count of Synthetic communities with diverging initial evenness, within transfer by Flow cytometric fingerprinting (FCFP) analysis.

Transfer	Evenness (mean ± SEM)			*P* value
	High	Low	Medium	
	Diversity (D_2)			
1	2308.73 ^{a} ± 29.36	2365.91 ^{a} ± 32.25	2483.41 ^{b} ± 40.99	0.0027
2	1916.85 ^{a} ± 25.28	2332.4 ^{b} ± 25.28	2393.11 ^{b} ± 35.30	<.0001
3	1797.91 ± 25.92	2466.2 ± 26.09	2238.78 ± 36.54	<.0001
4	1773.93 ± 21.31	2442.84 ± 21.59	2087.42 ± 30.05	<.0001
5	1703.76 ± 25.14	2447.54 ± 25.71	2022.04 ± 35.67	<.0001
	Total cell count			
1	5392576^{b} ± 135523	780095^{a} ± 150832	5658515^{b} ± 252801	<0.0001
2	6349780 ± 134640	3467298 ± 136432	5361839 ± 251196	<0.0001
3	5962616 ± 133347	2718790 ± 135991	5058574 ± 251196	<0.0001
4	4983684 ± 133774	1816368 ± 136877	3905662 ± 249621	<0.0001
5	5358130 ± 132506	3037760 ± 137327	4425697 ± 251196	<0.0001

Supplementary Table S2: Diversity indices and relative abundances of bacteria in Synthetic communities at transfer 5.

Metric	Evenness group			
	Low	Medium	High	*P* value
Pielou	0.574	0.576	0.568	0.97
Shannon	1.026	1.046	1.026	0.94
Simpson	0.547	0.564	0.537	0.74
Fisher	0.739	0.731	0.726	0.92
Inverse Simpson	2.354	2.346	2.289	0.83
Total species	6.2	6.15	6.11	0.93
Delftia	51.8	54.24	56.97	0.39
Aeromonas	20.14	20.61	17.62	0.46
Serratia	7.737	11.743	13.725	0.08
Bacillus	8.620	6.898	4.051	0.66
Clostridium	8.805 ^{a}	3.485 ^{b}	2.368 ^{b}	<0.0001
Tissierella	1.076 ^{b}	2.016 ^{b}	4.819 ^{a}	0.001
Bacillus I	0.004	0.011	0.041	0.39
Rhizobium	0.004	0.000	0.013	0.55
Enterococcus	1.8202 ^{a}	1.0017 ^{ab}	0.3816 ^{b}	0.0007
Burkholderia	0.004	0.000	0.008	0.56

Supplementary table S3: Correlation between relative bacterial abundance and three different evenness groups, indicated by the Multiple Factor Analysis. Dimensions of the MFA can be described by the categorical variables included in the analysis. The continuous variables were the relative abundances of the bacterial species and the categorical variable was the initial evenness category. The *P* value associated to this test is transformed to a normal quantile to assess whether the mean of the category is significantly less or greater than 0. Negative values indicate negative correlations.

**Dimension**	**Variance**	**Descriptor**	**Estimate (R^2)**	**P value**	**Taxon**	**Correlation**	***P* value**
					Shannon	0.97	<0.0001
					Simpson	0.96	<0.0001
					Inv Simpson	0.93	<0.0001
					Pielou	0.9	<0.0001
					Clostridium	0.48	<0.0001
					Aeromonas	0.36	0.0002
					Tissierella	0.34	0.0007
1	28.3%	Low	0.81	0.77	*Delftia*	0.29	0.004
		High	-0.09	0.76	*Burkholderia*	0.23	0.022
					Enterococcus	0.23	0.026
					Serratia	0.2	0.043
					Bacillus I	0.2	0.044
					Rhizobium	0.2	0.044
					Fisher	0.18	0.068
					Total species	0.18	0.068
					Bacillus	-0.71	<0.0001
					Rhizobium	0.87	<0.0001
					Burkholderia	0.86	<0.0001
					Bacillus I	0.86	<0.0001
					Fisher	0.74	<0.0001
					Total species	0.73	<0.0001
					Bacillus	0.25	0.01
		High	0.23	0.43	*Serratia*	0.25	0.01
2	23.92%	Medium	-0.06	0.7	*Enterococcus*	0.03	0.78
		Low	-0.16	0.63	Inv Simpson	-0.03	0.75
					Shannon	-0.04	0.68
					Tissierella	-0.06	0.57
					Clostridium	-0.08	0.42
					Simpson	-0.11	0.29
					Delftia	-0.25	0.01
					Pielou	-0.3	0.003
					Aeromonas	-0.36	0.0002
		Low	0.1	0.55	*Serratia*	0.64	<0.0001
3	13.63%	Medium	0.04	0.87	*Enterococcus*	0.5	<0.0001
		High	-0.14	0.45	*Bacillus*	0.4	<0.0001

					Inv Simpson	0.21	0.35
					Shannon	0.17	0.09
					Pielou	0.14	0.15
					Simpson	0.11	0.25
					Tissierella	0.09	0.39
					Total species	0.03	0.75
					Fisher	0.03	0.79
					Clostridium	-0.02	0.85
					Rhizobium	-0.23	0.03
					Bacillus I	-0.26	0.009
					Burkholderia	-0.27	0.008
					Delftia	-0.64	<0.0001
					Aeromonas	-0.68	<0.0001
					Tissierella	0.73	<0.0001
					Total species	0.44	<0.0001
					Fisher	0.43	<0.0001
					Delftia	0.12	0.23
					Shannon	0.1	0.32
					Bacillus	-0.04	0.68
4	10%	High<br>Medium<br>Low	0.48<br>0.09<br>-0.6	0.001<br>0.59<br>0.0002	*Aeromonas*	-0.04	0.67
					Serratia	-0.05	0.61
					Pielou	-0.08	0.38
					Bacillus I	-0.23	0.04
					Rhizobium	-0.25	0.01
					Burkholderia	-0.26	0.01
					Enterococcus	-0.37	0.0001
					Clostridium	-0.5	<0.0001
					Clostridium	0.58	<0.0001
					Total species	0.41	<0.0001
					Fisher	0.41	<0.0001
					Bacillus	0.19	0.06
					Enterococcus	0.17	0.1
					Aeromonas	0.16	0.11
5	7.98%	Low<br>Medium<br>High	0.76<br>-0.07<br>-0.69	<0.0001<br>0.61<br><0.0001	Inv Simpson	0.08	0.41
					Pielou	-0.14	0.16
					Burkholderia	-0.14	0.16
					Delftia	-0.17	0.1
					Tissierella	-0.19	0.05
					Bacillus I	-0.23	0.02
					Rhizobium	-0.23	0.02
					Serratia	-0.48	<0.0001

Supplementary table S4: Design overview of all 100 Synthetic community assemblies. Nr represents the number of each assembly, b1-b10 represent the 10 strains from Table 2 in the manuscript. Evenness refers to Pielou evenness and total Cell count sums b1-b10 and is expressed in cells/mL.

Nr	b1	b2	b3	b4	b5	b6	b7	b8	b9	b10	evenness	Cell count
**1**	1,88E+06	1,88E+03	9,38E+02	1,88E+03	1,88E+03	9,38E+02	1,88E+03	9,38E+02	9,38E+02	1,88E+03	0,024481	1,89E+06
**2**	7,89E+02	1,58E+03	1,58E+06	1,58E+03	1,58E+03	1,58E+03	1,58E+03	1,58E+03	1,58E+03	1,58E+03	0,02911	1,59E+06
**3**	1,15E+03	1,15E+04	1,15E+03	2,31E+06	1,15E+03	2,31E+03	1,15E+03	1,15E+03	1,15E+03	2,31E+03	0,031458	2,33E+06
**4**	1,15E+03	1,15E+04	2,31E+03	2,31E+06	1,15E+03	2,31E+03	1,15E+03	1,15E+03	1,15E+03	1,15E+03	0,031458	2,33E+06
**5**	1,15E+04	2,31E+06	1,15E+03	1,15E+03	2,31E+03	1,15E+03	1,15E+03	1,15E+03	2,31E+03	1,15E+03	0,031458	2,33E+06
**6**	1,07E+03	1,07E+04	1,07E+03	1,07E+03	2,14E+06	2,14E+03	2,14E+03	1,07E+03	1,07E+03	2,14E+03	0,032995	2,17E+06
**7**	1,07E+04	1,07E+03	2,14E+06	2,14E+03	2,14E+03	2,14E+03	1,07E+03	1,07E+03	1,07E+03	1,07E+03	0,032995	2,17E+06
**8**	1,07E+03	2,14E+03	2,14E+03	1,07E+03	1,07E+03	1,07E+03	1,07E+04	1,07E+03	2,14E+03	2,14E+06	0,032995	2,17E+06
**9**	1,07E+03	1,07E+03	1,07E+03	2,14E+03	2,14E+03	2,14E+03	2,14E+06	1,07E+03	1,07E+03	1,07E+04	0,032995	2,17E+06
**10**	1,07E+03	1,07E+03	1,07E+03	2,14E+03	2,14E+03	2,14E+03	1,07E+03	1,07E+03	2,14E+06	1,07E+04	0,032995	2,17E+06
**11**	1,00E+04	1,00E+03	1,00E+03	2,00E+03	1,00E+03	2,00E+03	2,00E+03	2,00E+03	2,00E+06	1,00E+03	0,034531	2,02E+06
**12**	2,00E+03	2,00E+03	2,00E+03	2,00E+03	1,00E+03	1,00E+03	1,00E+03	1,00E+03	2,00E+06	1,00E+04	0,034531	2,02E+06
**13**	2,00E+03	2,00E+06	1,00E+03	1,00E+03	2,00E+03	1,00E+04	1,00E+03	2,00E+03	2,00E+03	1,00E+03	0,034531	2,02E+06
**14**	2,00E+03	2,00E+03	1,00E+03	1,00E+03	2,00E+03	1,00E+04	2,00E+03	2,00E+06	1,00E+03	1,00E+03	0,034531	2,02E+06
**15**	1,00E+03	2,00E+03	1,00E+03	1,00E+03	2,00E+03	2,00E+06	1,00E+04	1,00E+03	2,00E+03	2,00E+03	0,034531	2,02E+06
**16**	1,88E+03	9,38E+03	9,38E+02	1,88E+03	1,88E+03	1,88E+03	9,38E+02	1,88E+06	1,88E+03	9,38E+02	0,036065	1,90E+06
**17**	1,15E+03	2,31E+03	2,31E+03	1,15E+03	1,15E+06	1,15E+03	1,15E+03	1,15E+03	1,15E+03	2,31E+03	0,038969	1,17E+06
**18**	1,36E+03	1,36E+03	1,36E+03	1,36E+06	1,36E+03	1,36E+03	1,36E+03	1,36E+03	2,73E+03	1,36E+04	0,053707	1,39E+06
**19**	1,15E+03	2,31E+03	1,15E+03	1,15E+06	2,31E+03	1,15E+03	1,15E+03	1,15E+04	1,15E+03	2,31E+03	0,059167	1,18E+06
**20**	1,15E+03	2,31E+03	1,15E+03	1,15E+03	2,31E+03	1,15E+03	2,31E+03	1,15E+04	1,15E+06	1,15E+03	0,059167	1,18E+06
**21**	1,15E+06	1,15E+03	2,31E+03	1,15E+03	2,31E+03	1,15E+03	1,15E+03	2,31E+03	1,15E+03	1,15E+04	0,059167	1,18E+06
**22**	2,31E+03	1,15E+03	1,15E+03	2,31E+03	1,15E+03	2,31E+03	1,15E+03	1,15E+06	1,15E+04	1,15E+03	0,059167	1,18E+06
**23**	1,07E+03	2,14E+03	2,14E+03	2,14E+03	1,07E+04	1,07E+03	2,14E+03	1,07E+03	1,07E+03	1,07E+06	0,061889	1,10E+06

**24**	1,07E+03	2,14E+03	1,07E+04	1,07E+06	2,14E+03	1,07E+03	2,14E+03	1,07E+03	2,14E+03	1,07E+03	0,061889	1,10E+06
**25**	1,15E+03	1,15E+03	2,31E+03	2,31E+03	2,31E+05	1,15E+03	1,15E+03	1,15E+03	1,15E+03	1,15E+03	0,137505	2,43E+05
**26**	1,88E+03	9,38E+02	9,38E+02	9,38E+02	1,88E+03	1,88E+03	9,38E+02	1,88E+03	1,88E+05	1,88E+03	0,165852	2,01E+05
**27**	1,76E+05	1,76E+03	1,76E+03	1,76E+03	1,76E+03	1,76E+03	8,82E+02	8,82E+02	1,76E+03	8,82E+02	0,175144	1,90E+05
**28**	1,76E+03	1,76E+03	8,82E+02	1,76E+03	1,76E+03	1,76E+05	8,82E+02	1,76E+03	1,76E+03	8,82E+02	0,175144	1,90E+05
**29**	1,25E+03	1,25E+03	1,25E+04	1,25E+03	1,25E+03	1,25E+03	2,50E+03	1,25E+03	2,50E+05	1,25E+03	0,190636	2,74E+05
**30**	1,25E+03	1,25E+04	1,25E+03	2,50E+05	1,25E+03	2,50E+03	1,25E+03	1,25E+03	1,25E+03	1,25E+03	0,190636	2,74E+05
**31**	1,15E+03	2,31E+05	1,15E+03	1,15E+04	2,31E+03	1,15E+03	2,31E+03	1,15E+03	1,15E+03	1,15E+03	0,199649	2,54E+05
**32**	1,15E+03	1,15E+03	1,15E+04	2,31E+03	1,15E+03	2,31E+05	1,15E+03	2,31E+03	1,15E+03	1,15E+03	0,199649	2,54E+05
**33**	1,15E+04	2,31E+05	2,31E+03	1,15E+03	1,15E+03	2,31E+03	1,15E+03	1,15E+03	1,15E+03	1,15E+03	0,199649	2,54E+05
**34**	2,14E+03	2,14E+05	1,07E+03	1,07E+03	1,07E+03	1,07E+03	2,14E+03	2,14E+03	1,07E+04	1,07E+03	0,208591	2,37E+05
**35**	2,14E+03	1,07E+03	1,07E+03	2,14E+03	1,07E+03	2,14E+05	2,14E+03	1,07E+03	1,07E+03	1,07E+04	0,208591	2,37E+05
**36**	1,07E+04	2,14E+03	2,14E+03	1,07E+03	1,07E+03	1,07E+03	2,14E+05	2,14E+03	1,07E+03	1,07E+03	0,208591	2,37E+05
**37**	1,07E+04	1,07E+03	2,14E+03	2,14E+05	1,07E+03	2,14E+03	1,07E+03	1,07E+03	1,07E+03	2,14E+03	0,208591	2,37E+05
**38**	1,07E+03	2,14E+03	2,14E+03	1,07E+03	2,14E+05	2,14E+03	1,07E+03	1,07E+03	1,07E+03	1,07E+04	0,208591	2,37E+05
**39**	2,00E+05	1,00E+03	1,00E+03	2,00E+03	2,00E+03	1,00E+03	2,00E+03	1,00E+03	1,00E+04	2,00E+03	0,21746	2,22E+05
**40**	1,00E+03	1,00E+04	1,00E+03	1,00E+03	2,00E+03	1,00E+03	2,00E+05	2,00E+03	2,00E+03	2,00E+03	0,21746	2,22E+05
**41**	1,07E+04	2,14E+04	1,07E+05	1,07E+04	1,07E+04	2,14E+05	2,14E+03	2,14E+05	1,07E+03	1,07E+06	0,501516	1,66E+06
**42**	1,25E+03	1,25E+03	1,25E+05	1,25E+05	1,25E+05	1,25E+05	2,50E+03	1,25E+06	2,50E+03	1,25E+05	0,520983	1,88E+06
**43**	2,14E+05	2,14E+05	1,07E+03	2,14E+05	1,07E+05	1,07E+03	1,07E+03	2,14E+03	1,07E+03	1,07E+06	0,546621	1,83E+06
**44**	1,88E+03	1,88E+04	9,38E+02	9,38E+05	9,38E+04	9,38E+05	1,88E+04	1,88E+04	1,88E+05	1,88E+04	0,538311	2,23E+06
**45**	2,14E+05	1,07E+05	2,14E+03	2,14E+03	1,07E+05	1,07E+04	1,07E+05	2,14E+03	1,07E+03	1,07E+06	0,496732	1,63E+06
**46**	1,07E+06	1,07E+04	1,07E+05	2,14E+04	1,07E+04	1,07E+05	1,07E+06	2,14E+04	2,14E+04	2,14E+03	0,510523	2,45E+06
**47**	1,07E+06	2,14E+04	1,07E+05	2,14E+03	2,14E+05	1,07E+04	2,14E+03	1,07E+03	1,07E+03	1,07E+06	0,501422	2,50E+06
**48**	2,00E+04	2,00E+04	1,00E+03	1,00E+03	2,00E+05	2,00E+03	1,00E+06	1,00E+03	1,00E+06	2,00E+03	0,452871	2,25E+06
**49**	1,88E+03	1,88E+03	1,88E+04	9,38E+03	1,88E+05	9,38E+04	1,88E+04	1,88E+04	9,38E+05	9,38E+04	0,489073	1,38E+06
**50**	1,00E+03	1,00E+05	1,00E+06	2,00E+04	2,00E+05	1,00E+05	2,00E+04	2,00E+03	2,00E+03	1,00E+03	0,45377	1,45E+06
**51**	1,88E+03	1,88E+05	1,88E+03	1,88E+04	9,38E+03	1,88E+05	9,38E+05	1,88E+04	9,38E+03	9,38E+04	0,51287	1,47E+06
**52**	2,14E+03	2,14E+05	1,07E+06	2,14E+03	1,07E+04	1,07E+05	1,07E+05	2,14E+04	1,07E+03	1,07E+04	0,455764	1,55E+06
**53**	1,67E+03	1,67E+04	1,67E+05	1,67E+04	1,67E+04	1,67E+03	1,67E+03	8,33E+03	8,33E+02	1,67E+04	0,533044	2,48E+05

**54**	9,38E+02	9,38E+03	9,38E+04	1,88E+04	1,88E+05	1,88E+03	1,88E+03	1,88E+03	1,88E+03	9,38E+02	0,476671	3,19E+05
**55**	2,00E+03	1,00E+03	2,00E+03	1,00E+05	1,00E+03	1,00E+04	2,00E+05	1,00E+04	2,00E+04	2,00E+03	0,507002	3,48E+05
**56**	1,07E+03	1,07E+03	1,07E+05	1,07E+04	2,14E+03	1,07E+04	2,14E+03	2,14E+04	2,14E+05	1,07E+04	0,536195	3,81E+05
**57**	1,76E+03	1,76E+03	1,76E+03	1,76E+03	1,76E+05	8,82E+04	1,76E+05	8,82E+03	1,76E+03	8,82E+02	0,541363	4,60E+05
**58**	1,76E+03	1,76E+04	1,76E+04	1,76E+03	8,82E+04	8,82E+02	1,76E+05	1,76E+03	8,82E+02	1,76E+03	0,502195	3,09E+05
**59**	2,00E+03	2,00E+05	1,00E+03	1,00E+03	2,00E+04	2,00E+03	1,00E+05	2,00E+03	1,00E+03	1,00E+04	0,471106	3,39E+05
**60**	8,82E+03	1,76E+04	1,76E+03	1,76E+04	1,76E+05	8,82E+04	1,76E+03	1,76E+03	1,76E+03	8,82E+02	0,536556	3,17E+05
**61**	1,00E+05	1,00E+05	2,00E+05	2,00E+05	1,00E+05	2,00E+05	2,00E+05	1,00E+06	2,00E+05	1,00E+05	0,838115	2,40E+06
**62**	2,00E+05	1,00E+05	1,00E+05	2,00E+05	1,00E+05	1,00E+05	2,00E+05	2,00E+05	2,00E+05	1,00E+06	0,838115	2,40E+06
**63**	1,76E+04	8,82E+05	1,76E+05	1,76E+05	1,76E+05	1,76E+05	1,76E+05	8,82E+04	1,76E+05	8,82E+04	0,827098	2,14E+06
**64**	1,76E+05	1,76E+05	8,82E+04	8,82E+05	1,76E+05	8,82E+04	1,76E+05	1,76E+04	1,76E+05	1,76E+05	0,827098	2,14E+06
**65**	2,14E+05	1,07E+05	1,07E+05	2,14E+05	1,07E+05	1,07E+05	1,07E+06	2,14E+05	2,14E+05	1,07E+05	0,822239	2,46E+06
**66**	1,58E+05	1,58E+05	1,58E+05	1,58E+05	1,58E+04	7,89E+05	1,58E+05	1,58E+05	1,58E+04	1,58E+05	0,81629	1,93E+06
**67**	1,88E+05	9,38E+04	9,38E+04	1,88E+05	1,88E+05	9,38E+02	1,88E+05	9,38E+05	1,88E+05	1,88E+05	0,81428	2,25E+06
**68**	9,38E+04	9,38E+05	1,88E+05	1,88E+05	1,88E+05	9,38E+04	1,88E+05	1,88E+05	9,38E+02	1,88E+05	0,81428	2,25E+06
**69**	1,88E+05	9,38E+05	1,88E+05	9,38E+04	9,38E+02	1,88E+05	1,88E+05	9,38E+04	1,88E+05	1,88E+05	0,81428	2,25E+06
**70**	1,88E+04	9,38E+04	1,88E+05	1,88E+05	1,88E+05	1,88E+05	1,88E+05	9,38E+04	9,38E+05	9,38E+04	0,810725	2,18E+06
**71**	1,88E+05	1,88E+05	1,88E+05	1,88E+05	9,38E+05	1,88E+05	1,88E+04	9,38E+04	9,38E+04	9,38E+04	0,810725	2,18E+06
**72**	1,88E+05	9,38E+04	1,88E+05	1,88E+04	1,88E+05	9,38E+05	1,88E+05	9,38E+04	1,88E+05	9,38E+04	0,810725	2,18E+06
**73**	9,38E+05	1,88E+05	9,38E+04	1,88E+05	9,38E+04	9,38E+04	1,88E+04	1,88E+05	1,88E+05	1,88E+05	0,810725	2,18E+06
**74**	9,38E+04	9,38E+04	1,88E+05	1,88E+05	1,88E+05	1,88E+05	1,88E+05	9,38E+04	9,38E+05	1,88E+04	0,810725	2,18E+06
**75**	1,88E+04	1,88E+05	1,88E+05	9,38E+04	1,88E+05	1,88E+05	9,38E+04	9,38E+04	1,88E+05	9,38E+05	0,810725	2,18E+06
**76**	1,67E+05	8,33E+05	1,67E+05	1,67E+05	1,67E+04	1,67E+05	1,67E+05	1,67E+05	8,33E+03	1,67E+05	0,810519	2,03E+06
**77**	1,58E+05	1,58E+05	1,58E+05	7,89E+04	1,58E+05	1,58E+05	1,58E+05	1,58E+05	1,58E+05	1,58E+05	0,993567	1,50E+06
**78**	1,67E+05	1,67E+05	8,33E+04	1,67E+05	1,67E+05	1,67E+05	1,67E+05	1,67E+05	8,33E+04	1,67E+04	0,957995	1,35E+06
**79**	8,33E+04	1,67E+05	1,67E+05	1,67E+05	1,67E+05	8,33E+04	1,67E+05	1,67E+05	1,67E+05	1,67E+04	0,957995	1,35E+06
**80**	1,15E+05	2,31E+05	1,15E+05	1,15E+05	2,31E+05	1,15E+05	1,15E+05	1,15E+05	1,15E+05	2,31E+04	0,954189	1,29E+06
**81**	1,07E+05	1,07E+05	2,14E+05	1,07E+05	2,14E+04	2,14E+05	1,07E+05	2,14E+05	1,07E+05	1,07E+05	0,949771	1,31E+06
**82**	2,14E+05	2,14E+05	1,07E+05	1,07E+05	1,07E+05	2,14E+04	1,07E+05	1,07E+05	2,14E+05	1,07E+05	0,949771	1,31E+06
**83**	1,67E+05	1,67E+05	1,67E+05	1,67E+05	8,33E+02	1,67E+05	1,67E+05	1,67E+05	8,33E+04	1,67E+05	0,948724	1,42E+06

**84**	2,00E+04	1,00E+05	2,00E+05	1,00E+05	1,00E+05	2,00E+05	1,00E+05	1,00E+05	2,00E+05	2,00E+05	0,948722	1,32E+06
**85**	2,00E+04	1,00E+04	2,00E+04	1,00E+04	1,00E+04	2,00E+04	2,00E+04	2,00E+04	1,00E+04	1,00E+04	0,975405	1,50E+05
**86**	8,33E+03	1,67E+04	1,67E+04	1,67E+04	1,67E+04	1,67E+04	1,67E+04	1,67E+04	1,67E+03	8,33E+03	0,957995	1,35E+05
**87**	1,76E+04	1,76E+04	1,76E+04	1,76E+04	8,82E+03	1,76E+03	8,82E+03	1,76E+04	1,76E+04	8,82E+03	0,953385	1,34E+05
**88**	1,88E+04	1,88E+04	1,88E+04	1,88E+03	1,88E+04	1,88E+04	9,38E+03	9,38E+03	9,38E+03	9,38E+03	0,95014	1,33E+05
**89**	1,88E+03	1,88E+04	9,38E+03	1,88E+04	9,38E+03	9,38E+03	1,88E+04	1,88E+04	9,38E+03	1,88E+04	0,95014	1,33E+05
**90**	1,00E+04	1,00E+04	2,00E+04	2,00E+04	1,00E+04	2,00E+04	2,00E+03	2,00E+04	1,00E+04	1,00E+04	0,948722	1,32E+05
**91**	1,25E+04	1,25E+04	1,25E+04	1,25E+04	1,25E+03	1,25E+04	1,25E+04	2,50E+03	2,50E+04	1,25E+04	0,92953	1,16E+05
**92**	1,25E+04	1,25E+04	1,25E+04	2,50E+03	1,25E+04	1,25E+04	1,25E+04	1,25E+03	2,50E+04	1,25E+04	0,92953	1,16E+05
**93**	1,07E+04	2,14E+04	1,07E+04	1,07E+04	2,14E+03	1,07E+04	1,07E+04	2,14E+04	1,07E+04	2,14E+03	0,928136	1,11E+05
**94**	1,07E+04	2,14E+03	2,14E+03	1,07E+04	1,07E+04	1,07E+04	2,14E+04	1,07E+04	2,14E+04	1,07E+04	0,928136	1,11E+05
**95**	1,07E+04	1,07E+04	2,14E+03	2,14E+03	2,14E+04	1,07E+04	2,14E+04	1,07E+04	1,07E+04	1,07E+04	0,928136	1,11E+05
**96**	8,33E+03	8,33E+03	1,67E+04	1,67E+04	1,67E+03	1,67E+04	1,67E+04	1,67E+04	1,67E+03	1,67E+04	0,92692	1,20E+05
**97**	1,76E+03	8,82E+03	1,76E+04	8,82E+03	1,76E+04	1,76E+04	8,82E+03	1,76E+04	1,76E+04	1,76E+03	0,92332	1,18E+05
**98**	1,76E+03	1,76E+04	1,76E+03	1,76E+04	8,82E+03	1,76E+04	1,76E+04	8,82E+03	1,76E+04	8,82E+03	0,92332	1,18E+05
**99**	9,38E+03	9,38E+03	1,88E+03	1,88E+04	1,88E+04	9,38E+03	1,88E+03	1,88E+04	9,38E+03	1,88E+04	0,921756	1,16E+05
**100**	9,38E+03	1,88E+03	1,88E+04	9,38E+03	1,88E+03	9,38E+03	9,38E+03	1,88E+04	1,88E+04	1,88E+04	0,921756	1,16E+05

Supplementary table S5: Operational Taxonomic Units (OUT) table with around 200 Synthetic community assemblies (SynC) in transfer 0 and 5. OUT 1-10 represent the 10 strains from Table 2 in the manuscript.

SynC	transfer	OTU 1	OTU 2	OTU 3	OTU 4	OTU 5	OTU 6	OTU 7	OTU 8	OTU 9	OTU 10
SynC1	0	24	23	7	3	1006	344	1903	6	0	1
SynC2	0	73	15	3180	6	5	0	7	20	0	11
SynC3	0	1036	48	282	5	78	457	38	12	127	1234
SynC4	0	948	502	76	20	42	459	67	29	26	1148
SynC5	0	1438	752	30	3	451	0	589	10	42	2
SynC6	0	1391	16	431	942	210	0	0	4	321	2
SynC7	0	1132	7	1759	2	255	3	6	2	150	1
SynC8	0	947	1718	196	1	421	0	2	0	32	0
SynC9	0	1200	994	416	4	502	0	2	2	196	1
SynC10	0	2353	222	271	7	256	0	2	5	201	0
SynC11	0	2232	32	544	12	236	17	40	16	188	0
SynC12	0	2175	547	32	4	436	0	16	26	81	0
SynC13	0	498	578	149	1786	12	1	262	13	13	5
SynC14	0	509	413	45	825	14	1	12	1483	8	7

SynC15	0	850	1046	118	17	204	0	11	9	1057	5
SynC16	0	953	275	337	15	288	2	10	1291	145	1
SynC17	0	771	1020	270	940	296	0	5	6	6	3
SynC18	0	1050	1528	79	2	74	4	3	1	91	485
SynC19	0	1145	787	748	8	6	0	5	86	57	475
SynC20	0	1554	69	1385	97	14	4	11	152	31	0
SynC21	0	1006	1012	95	0	796	53	342	3	8	2
SynC22	0	1346	1064	184	1	6	2	5	679	27	3
SynC23	0	948	1620	169	31	489	0	10	7	38	5
SynC24	0	763	1068	404	6	520	0	5	3	25	523
SynC25	0	1036	1167	218	376	496	0	3	9	9	3
SynC26	0	2147	662	53	0	423	0	4	14	5	9
SynC27	0	1207	711	56	4	615	51	641	0	30	2
SynC28	0	1338	765	109	15	524	4	32	17	499	14
SynC29	0	2397	664	162	2	73	0	5	4	10	0
SynC30	0	1150	739	190	23	384	2	39	19	31	740
SynC31	0	1555	820	132	11	514	0	100	12	102	71
SynC32	0	1014	42	1436	11	157	0	16	15	621	5
SynC33	0	1781	983	216	8	66	20	187	26	30	0

SynC34	0	1694	1035	238	15	15	6	164	66	84	0
SynC35	0	110	1360	100	67	53	11	105	64	1447	0
SynC36	0	214	309	423	174	853	64	1076	185	19	0
SynC37	0	139	327	225	51	131	14	285	88	13	2044
SynC38	0	78	1113	116	1951	7	1	8	31	12	0
SynC39	0	360	87	78	27	1109	273	1362	19	2	0
SynC40	0	518	1019	547	613	108	6	248	233	25	0
SynC41	0	11	2367	339	59	19	1	28	403	84	6
SynC42	0	8	799	491	429	2	0	1	1449	47	91
SynC43	0	10	2191	4	305	231	58	368	7	0	143
SynC44	0	767	356	18	616	6	1	12	143	483	915
SynC45	0	7	2190	10	291	295	106	407	8	2	1
SynC46	0	76	15	594	25	913	253	1370	38	24	9
SynC47	0	33	1766	224	196	351	161	577	2	7	0
SynC48	0	2379	50	17	682	72	11	98	6	0	2
SynC49	0	1623	750	167	511	3	1	11	62	175	14
SynC50	0	34	38	2164	678	4	0	10	6	281	102
SynC51	0	547	1524	72	78	6	0	507	103	321	159
SynC52	0	40	85	2572	49	1	3	292	165	100	10

SynC53	0	73	399	1991	268	7	0	141	171	2	265
SynC54	0	123	27	1690	1106	6	2	32	42	2	287
SynC55	0	1497	141	154	29	19	5	75	163	35	1199
SynC56	0	1863	160	1030	15	1	0	2	202	12	32
SynC57	0	475	926	380	1348	11	1	14	57	96	9
SynC58	0	72	1233	1396	570	7	0	16	17	1	5
SynC59	0	50	3133	43	49	1	3	30	3	0	5
SynC60	0	352	983	538	932	43	12	72	30	211	144
SynC61	0	851	365	606	169	65	8	167	783	57	246
SynC62	0	1156	1055	517	66	104	7	129	170	13	100
SynC63	0	689	2053	300	69	4	0	24	86	3	89
SynC64	0	555	2233	101	38	36	3	34	12	3	302
SynC65	0	825	1783	248	66	76	9	94	119	4	93
SynC66	0	271	1889	529	20	39	23	156	105	284	1
SynC67	0	512	2225	92	58	39	2	46	272	1	70
SynC68	0	49	1039	695	474	77	42	240	299	48	354
SynC69	0	688	995	630	0	157	81	419	144	76	127
SynC70	0	1136	811	470	372	19	4	38	135	101	231
SynC71	0	489	1701	475	294	73	9	89	63	19	105

SynC72	0	781	1940	304	85	47	5	50	84	11	10
SynC73	0	595	1761	180	40	286	25	235	89	3	103
SynC74	0	1303	137	752	317	101	51	134	182	75	265
SynC75	0	1118	907	873	134	11	3	26	78	84	83
SynC76	0	85	952	1071	40	91	47	317	298	96	320
SynC77	0	1229	748	568	134	157	12	124	209	31	105
SynC78	0	1023	180	667	199	207	30	299	298	168	246
SynC79	0	819	1260	714	123	4	9	219	46	123	0
SynC80	0	1419	109	1090	162	60	3	89	170	17	198
SynC81	0	887	593	957	23	73	4	205	245	158	172
SynC82	0	1434	588	550	72	116	26	302	106	17	106
SynC83	0	804	785	709	1	122	20	325	206	108	237
SynC84	0	1200	909	725	89	11	0	48	94	146	95
SynC86	0	274	925	1562	94	29	5	85	119	73	151
SynC87	0	866	759	1256	37	33	6	93	135	7	125
SynC88	0	647	866	1457	71	45	5	135	33	50	8
SynC89	0	568	1475	877	170	6	1	16	64	29	111
SynC90	0	716	887	1116	63	34	2	74	125	121	179
SynC91	0	1266	533	942	9	79	13	198	36	102	139

SynC92	0	1304	518	1049	98	52	17	180	15	59	25
SynC93	0	1124	457	986	20	51	4	144	302	73	156
SynC94	0	970	1946	162	55	32	6	61	48	13	24
SynC95	0	746	2155	154	103	23	2	61	45	14	14
SynC96	0	149	2400	541	9	22	10	46	48	24	68
SynC97	0	775	1081	1147	82	14	0	11	113	59	35
SynC98	0	909	871	382	367	17	8	69	278	97	319
SynC99	0	536	1041	208	508	98	22	179	407	47	271
SynC100	0	757	610	1073	64	90	32	214	309	41	127
SynC1	5	1931	470	274	0	78	475	0	0	90	0
SynC2	5	1990	527	343	0	284	0	0	0	174	0
SynC3	5	1768	340	563	1	500	0	1	1	145	0
SynC4	5	1823	1	605	0	503	0	0	0	386	1
SynC5	5	1945	316	500	0	403	0	0	1	155	0
SynC6	5	544	45	6	2704	13	1	0	2	4	1
SynC7	5	1852	589	308	3	409	1	0	0	156	1
SynC8	5	1933	958	72	0	287	4	0	0	65	0
SynC9	5	894	168	2135	43	3	0	0	1	75	0
SynC10	5	1762	970	211	0	336	0	0	0	39	0

SynC11	5	1634	863	77	0	686	0	0	0	58	1
SynC12	5	1777	909	76	0	542	0	0	0	14	0
SynC13	5	1971	979	30	1	309	0	1	0	28	0
SynC14	5	2076	906	95	0	228	0	0	0	13	0
SynC15	5	1698	421	776	1	287	1	0	1	135	0
SynC16	5	2106	1004	146	2	1	0	0	0	59	1
SynC17	5	93	36	5	3177	2	3	3	0	0	0
SynC18	5	1687	1041	172	1	400	0	0	0	18	1
SynC19	5	1521	705	708	0	313	0	0	0	71	0
SynC20	5	1714	1041	323	0	208	0	0	1	31	0
SynC21	5	1497	898	148	1	366	404	1	0	4	1
SynC22	5	1866	1140	36	0	260	1	1	0	16	0
SynC23	5	1789	1358	120	0	0	0	0	0	50	0
SynC24	5	1720	946	20	0	630	0	0	0	1	0
SynC25	5	701	36	1	2579	2	0	0	0	0	0
SynC26	5	1709	992	174	0	438	0	0	0	6	0
SynC27	5	1533	802	240	0	662	0	0	0	81	0
SynC28	5	1857	628	535	0	154	0	0	0	144	0
SynC29	5	1506	1033	34	0	719	0	0	0	26	0

SynC30	5	1983	948	35	1	337	1	0	0	14	0
SynC31	5	2198	960	34	1	113	1	0	0	12	0
SynC32	5	1966	892	44	1	406	0	0	0	10	1
SynC33	5	2036	832	82	0	334	0	0	0	34	0
SynC34	5	1789	911	56	0	531	0	0	0	30	1
SynC35	5	1995	1086	9	0	207	5	0	0	16	0
SynC36	5	2655	0	380	3	221	0	0	0	60	0
SynC37	5	2190	270	407	3	160	203	0	0	86	0
SynC38	5	353	38	5	2920	2	0	0	0	1	0
SynC39	5	1911	392	298	1	332	331	1	0	54	0
SynC40	5	2752	281	193	0	27	0	0	0	65	0
SynC41	5	1978	912	33	0	381	4	0	0	11	0
SynC42	5	2114	871	71	0	252	0	0	0	10	0
SynC43	5	2050	867	52	1	240	105	0	0	4	0
SynC44	5	2153	886	54	0	214	2	0	0	10	0
SynC45	5	1789	916	33	0	242	332	0	0	6	0
SynC46	5	1664	862	27	2	320	436	0	0	9	0
SynC47	5	2098	812	217	0	42	139	0	0	10	0
SynC48	5	2084	903	162	0	41	117	0	0	10	0

SynC49	5	2035	1226	32	0	14	0	0	0	11	0
SynC50	5	1939	932	400	0	24	0	0	0	24	0
SynC51	5	1862	867	526	0	7	1	0	0	55	0
SynC52	5	1654	524	898	0	136	64	7	0	35	0
SynC53	5	1718	459	584	396	92	48	0	0	22	0
SynC54	5	1500	38	1478	2	69	8	0	0	225	0
SynC55	5	2049	606	563	10	49	7	0	0	34	0
SynC56	5	1826	852	397	197	21	0	0	0	26	0
SynC57	5	42	4	763	2414	0	0	0	0	96	0
SynC58	5	2275	402	541	0	76	18	0	0	7	0
SynC59	5	2146	613	458	0	43	50	0	0	9	0
SynC60	5	1008	126	507	1557	55	11	0	0	54	0
SynC61	5	1899	410	737	0	53	202	0	0	18	0
SynC62	5	2566	394	150	0	31	166	0	0	12	0
SynC63	5	2208	880	172	0	43	1	0	0	15	0
SynC64	5	2412	579	189	0	30	98	0	0	11	0
SynC65	5	1908	706	426	0	72	197	0	0	8	0
SynC66	5	1973	1051	229	0	52	1	0	0	13	0
SynC67	5	1850	673	406	0	62	303	0	0	24	0

SynC68	5	1743	898	251	0	49	356	0	0	21	0
SynC69	5	1784	638	402	0	66	410	0	0	18	0
SynC70	5	1675	818	302	0	36	471	4	0	13	0
SynC71	5	1710	691	360	0	66	477	0	0	14	0
SynC72	5	1832	514	407	0	69	482	0	0	15	0
SynC73	5	1942	703	218	0	62	374	1	0	20	0
SynC74	5	1738	579	524	0	58	414	0	0	6	0
SynC75	5	2135	661	438	0	74	1	0	0	10	0
SynC76	5	1835	121	1207	0	44	88	0	0	24	0
SynC77	5	1872	668	419	0	87	270	0	0	3	0
SynC78	5	2031	608	270	0	36	360	0	0	13	0
SynC79	5	1966	468	524	0	50	289	0	0	21	0
SynC80	5	1857	260	681	0	43	470	0	0	7	0
SynC81	5	2075	739	195	0	43	261	0	0	6	0
SynC82	5	1969	1049	218	1	77	0	0	0	5	0
SynC86	5	2735	332	151	0	24	67	1	0	9	0
SynC87	5	2891	392	27	5	2	0	0	0	0	0
SynC88	5	2174	734	312	0	82	0	0	0	16	0
SynC89	5	1828	1345	122	0	18	0	0	0	4	0

SynC91	5	2657	515	104	5	19	1	5	0	12	0
SynC92	5	2263	719	265	1	43	0	0	0	26	1
SynC93	5	1807	408	717	1	375	0	0	0	10	0
SynC94	5	1902	547	665	0	186	0	0	0	19	0
SynC95	5	2152	470	567	0	124	0	0	0	5	0
SynC96	5	1880	689	522	0	221	0	1	0	6	0
SynC97	5	33	8	11	3242	0	0	0	0	24	0
SynC98	5	1003	357	1954	3	0	0	0	1	2	0
SynC99	5	59	7	1380	1566	289	0	0	0	17	0
SynC100	5	1679	418	876	15	251	2	39	15	17	9
